# Anti-Biofilm Compounds Derived from Marine Sponges

**DOI:** 10.3390/md9102010

**Published:** 2011-10-19

**Authors:** Sean D. Stowe, Justin J. Richards, Ashley T. Tucker, Richele Thompson, Christian Melander, John Cavanagh

**Affiliations:** 1 Department of Molecular & Structural Biochemistry, North Carolina State University, Raleigh, NC 27695, USA; E-Mails: sdstowe@gmail.com (S.D.S.); attucker@ncsu.edu (A.T.T.); richele_thompson@ncsu.edu (R.T.); 2 Department of Chemistry, North Carolina State University, Raleigh, NC 27695, USA; E-Mails: jjrichar@email.unc.edu (J.J.R.); ccmeland@ncsu.edu (C.M.)

**Keywords:** biofilms, antifouling, ageloxime-D, manoalide, 2-aminoimidazole

## Abstract

Bacterial biofilms are surface-attached communities of microorganisms that are protected by an extracellular matrix of biomolecules. In the biofilm state, bacteria are significantly more resistant to external assault, including attack by antibiotics. In their native environment, bacterial biofilms underpin costly biofouling that wreaks havoc on shipping, utilities, and offshore industry. Within a host environment, they are insensitive to antiseptics and basic host immune responses. It is estimated that up to 80% of all microbial infections are biofilm-based. Biofilm infections of indwelling medical devices are of particular concern, since once the device is colonized, infection is almost impossible to eliminate. Given the prominence of biofilms in infectious diseases, there is a notable effort towards developing small, synthetically available molecules that will modulate bacterial biofilm development and maintenance. Here, we highlight the development of small molecules that inhibit and/or disperse bacterial biofilms specifically through non-microbicidal mechanisms. Importantly, we discuss several sets of compounds derived from marine sponges that we are developing in our labs to address the persistent biofilm problem. We will discuss: discovery/synthesis of natural products and their analogues—including our marine sponge-derived compounds and initial adjuvant activity and toxicological screening of our novel anti-biofilm compounds.

## 1. Introduction

Biofouling is a costly and destructive natural phenomenon that affects almost every economic sector from shipping to medicine, causing billions of dollars in damage and disruptions annually. While biofouling is typically linked to aquatic invertebrates, it is the formation of a biofilm that acts as the glue that binds these animals to a surface [1–3]. Biofilms can be generated by numerous species of microorganisms but are primarily the creation of bacterial microcolonies that have attached to a surface and shielded themselves in an extracellular matrix of polysaccharide, protein, and nucleic acids [3–6]. Utilizing this line of defense, bacteria have been able to successfully permeate every environmental niche, including the human body [5–9]. According to the NIH, biofilm-based microbial infections make up to 80% of all infections in human patients, leading the CDC to declare biofilms to be one of the most important medical hurdles of the century [7,10].

In an effort to find viable sources of anti-biofilm agents, many researchers have started to extract and analyze natural products from a myriad of plants and marine organisms [11–13]. Many of these compounds are secondary metabolites that are generated by the host organism in response to external pressures, such as competition for space and potential predators [14–17]. In a marine environment, antimicrobial and antifouling metabolites are vital for many sessile organisms to insure that they do not host hazardous biofilms on their exposed surfaces, especially given that an overwhelming majority of the planet’s microbial biomass prefers to be in a biofilm state [6,18–20]. Of all the species studied, marine sponges (Phylum Porifera) have been some of the most valuable. Sponges have been the source of more than 30% of marine natural products, generating a diverse array of molecules that have been found to have not only antimicrobial capabilities but also have potential as cancer therapeutics [13,21].

Despite their potential, there are very few of these metabolites and their derivatives that can serve as biofilm modulators in a non-microbicidal manner. The purpose of this review is to present those compounds derived from sponges that have shown anti-biofilm potential without bactericidal effects, with particular focus on the screening and development of our own chemical libraries as an example. These non-lethal molecules are significant discoveries, because they have the potential to serve as therapeutic supplements that can enhance the efficiency of conventional antibiotics against biofilm-based infections without eliciting resistant phenotypes. While it is important to continue to expand our libraries of antibiotic molecules, it is essential for the continued efficacy of all antimicrobial drugs that new and effective adjuvants be discovered in order to mitigate the increasing microbial resistance derived from biofilms [7–9,18,22,23].

### 1.1. Biofilm Development and Defense

**Figure 1:**
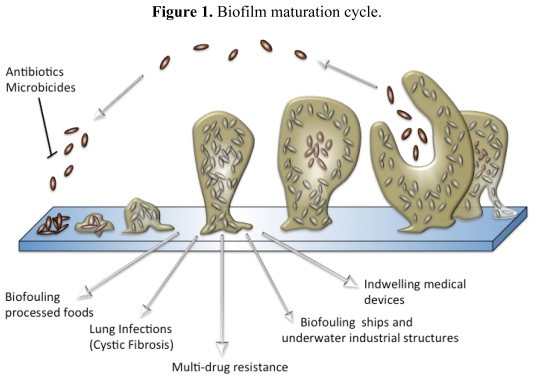
Biofilm maturation cycle.

Ever since van Leeuwenhoek noted his belief that the number of organisms found in 17th century dental plaque exceeded “the number of men in a kingdom”, biofilms have been a part of our understanding of how bacteria interact with their environment, but it was not until the late 20th century that we began to realize their true importance. Over the last few decades, intense study of a deluge of bacterial species from diverse environmental niches has lead to a generalized conception of how biofilms are formed [23,24]. For most species, biofilm formation occurs in five distinct stages (Figure 1) that are defined by a combination of phenotype and genetic changes [23]. Initially, planktonic bacteria reversibly attach themselves to surfaces that they encounter and remain in this transient state until signaled by an environmental cue to form a less ephemeral relationship. Every species of bacteria has a specific set of environmental signals that will initiate biofilm formation. Many of these signals are the chemical messages used for intercellular signaling within a population to coordinate altered gene expression in response to population density, a phenomenon known as quorum sensing [25–28]. It is also during the first stage of attachment that most antifouling and antimicrobial compounds are the most effective, because the bacteria have not coated themselves in the exopolymeric substance (EPS) and are in a vulnerable state.

**Figure 2:**
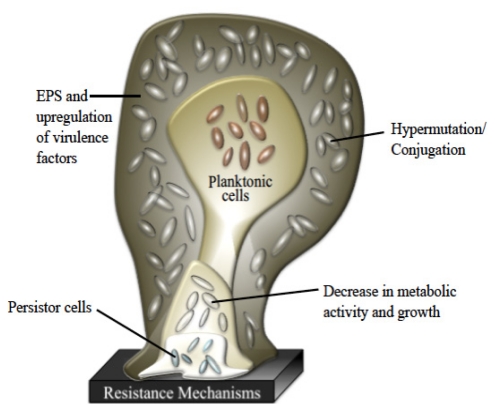
Various levels of defense provided by biofilms.

Once the bacteria begin to secrete EPS, biofilm development has progressed to its second stage and has become an irreversible process. The EPS is a complex, highly polar mixture of biomolecular materials that provides the resident bacteria with protection against bactericidal compounds and host immune responses [29]. EPS secretion continues throughout the third stage of formation, securely attached the bacteria to the surface within a thick, complex layer of biomolecules [30]. As the biofilm reaches maturity, it will take on a three-dimensional tower structure. Within these towers, small transport channels form, which facilitate the distribution of nutrients, water, and waste and carry planktonic bacteria to be housed in small cavities. In addition to logistical functions, the morphology of a biofilm lends itself as an ideal environment for hypermutation and increased gene transfer, which lead to higher rates of resistance [19,31–33]. Further exacerbating the resistance problem, bacterial metabolic activity within a biofilm is not uniform and highly dependent on the internal location of the bacterium, making the cell more able to withstand microbicides (Figure 2) [34–36]. Finally, once the biofilm reaches full maturity, sections of the tower begin to detach either by erosion (small loses) or by sloughing (large portions), and those cavities of free-floating bacteria are emptied, releasing fresh bacteria into the environment [37,38].

While physical characteristics of biofilm morphology contribute to the significant difference between planktonic and biofilm antibiotic resistance, no one has been able to discern a shared means of resistance for all species. One way of better defining this problem is the prevailing persistor cell theory. According to this theory, each biofilm contains a small population of highly resistant bacteria that are able to survive antibiotic kill-off to rebuild the biofilm population [39–41]. Although the concept of persistor cells has been around over seventy years, it has only been through the recent increase in medical biofilm research that the correlation between biofilm-based disease relapse models and persistor cells could have been made [42]. Since the persistor cells are protected, they are able to reproduce once the microbicidal insult abates and yield stronger progeny that have a significantly higher chance of being antibiotic resistant after each treatment, leading to a chronic disease that is driven by antibiotic resistant bacteria found in normal acute infections.

### 1.2. Biofilm Importance in Industry and Medicine

Over the last century, biofilm research has been primarily focused on their adverse role in industrial biofouling. Biofouling is the term used to describe the unwanted formation of microbial stratum or deposits of their waste products on a submerged surface [43]. The effects of biofouling can be felt across numerous industries and is a particular costly problem in food processing, utilities and marine-based industries. For most of the industrial victims, biofilm costs are directly related to a decrease in their production efficiency through energy losses, physical deterioration, and chemical interference [4,43–45]. A long-standing problem for naval and merchant ships has been the increased hydrodynamic drag and decreased maneuverability that is caused by biofouling of ship hulls, which increases fuel consumption and voyage costs [44]. Another costly result of biofouling is the long-term damage that results from the biocorrosion of equipment and other metal surfaces. Biocorrosion is a synergistic phenomenon that results from the combination of natural abiotic corrosion and biofilm-generated corrosive metabolites and waste products [4]. In an attempt to reduce the financial losses, many different antifouling strategies have been developed, especially for seafaring ships. Most of these antifouling techniques consist of coating the submerged surface with a chemically enhanced paint that prevents the successful attachment of fouling organisms [44]. Unfortunately, the most successful coatings relied on toxic heavy metals or organotins (tributyltin or TBT) that were banned due to their mutagenic effects and bioaccumulation in many marine animals [44,46–49]. Other antifouling methods have looked to use biocides or low energy polymers. Neither of these methods have been as successful as TBT at controlling fouling, and several of the “booster biocides” have been found to accumulate in marine environments [44,49]. While many marine natural products have proven to be good non-toxic antifouling agents, they are difficult to generate for large-scale uses, such as antifouling coatings. A promising solution to this production problem is the creation of antifouling agents that are biomimetic compounds or compounds that are synthetic in nature but inspired by natural products, many of which have been derived from marine sponge metabolites [50].

More recently, bacterial biofilms have been linked to a wide range of chronic ailments and are believed to be the principle culprits involved in persistent nosocomial infections that develop in immune-compromised patients [7,51,52]. By definition, biofilms provide their inhabitants with protection against many of the environmental and chemical threats that would normally destroy their planktonic brethren including many of the antiseptics and anti-microbial agents used to sterilize hospital facilities. This level of protection is also extended to protect invading bacteria against the host’s immune defenses and has been shown to increase bacterial resistance to conventional antibiotics by nearly 1000-fold [53–55].

Some of the worse cases of persistent biofilm-based infections occur when a patient receives an infected indwelling medical device (IMD), such as a catheter [56–58]. In many of these cases, the infection cannot be overcome without additional surgery to clean out the effected area and replace the tainted device with a new one [59]. Just as with biofouling in industry, medical research has looked to antifouling coatings to counter IMD infections. A wide range of antibiotic (e.g., cefazolin and rifampin/minocycline) and chemical compounds (e.g., salicylic acid and silver/chlorohexidine) have been tested as potential coatings, but many of these compounds have an inherent risk of causing resistance phenotypes to develop [56,60–67]. Despite significant advances in anti-microbial research in the last fifty years, there have only been two major antibiotic discoveries, oxazolidinones and lipopeptides [68,69]. Further exasperating this problem, these new classes of antibiotics are only active against Gram-positive infections. Therefore, the pursuit of compounds that have the ability to modulate biofilm formation has become a major goal for therapeutic research in order to overcome MDR infections [9].

## 2. Anti-Biofilm Agents Derived from Marine Sponges

Marine sponges can be likened to little factories for bioactive secondary metabolites. These benthic organisms are some of the simplest multicellular animals with little differentiation and long lives, relying on the water around them to supply all their essential needs. Therefore, the generation of chemical defenses is a key element of their survival [70], whether they need to ward off predators [71–73], fight off competition for space and resources [74–76] or control surface fouling [48,73]. Sponges utilize a plethora of chemical classes to protect themselves and even to communicate with symbiotic organisms that can provide nutrients and additional protection [77]. Many of these chemicals have been found to have antifouling and anti-biofilm properties, but very few have been shown to modulate biofilm formation without killing the bacteria or disrupting their growth. To date, only two classes of marine sponge metabolites house non-bactericidal biofilm modulators, the terpenoids [78,79] and the pyrrole-imidazoles [80–83].

Although not unique to sponges, one of the most potent and diverse groups of molecules is the terpenes and their derivatives. Terpenes have a high degree of structural diversity stemming from the modification of isoprene subunits and are valued for their broad range of biological activity, from antifouling agents to antiproliferative cancer therapeutics [16,84,85]. Within the sponge terpenoids, only ageloxime-D, manoalide, and two manoalide congeners have been reported to have the ability to infer with bacterial biofilm formation without disrupting cellular growth [78,79].

**Figure 3:**
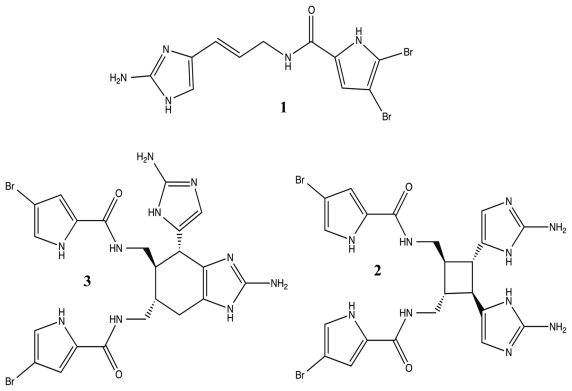
Potent antifouling pyrrole-imidazole alkaloid natural products: oroidin (**1**), sceptrin (**2**), and bromoageliferin (**3**).

In addition to the use of terpenoids, marine sponges are capable of synthesizing a class of potent molecules that is completely unique to their phylum, the pyrrole-imidazole alkaloids (PIA) [86]. PIAs have been extracted from several families of sponges with extensive focus of the bromopyrrole derivatives from the *Agelasidae* family [72,87,88]. As natural products, the PIAs, especially oroidin **1**, sceptrin **2**, and bromoageliferin **3** (Figure 3), have been found to be potent and toxic antifouling agents, working against microorganisms and higher organisms alike [72,86,87,89]. Yet, from these molecules, the Melander group has been able to construct a series of very successful anti-biofilm libraries that utilize the 2-aminoimidazole moiety found within many of the PIAs [49,80,83,86,90–93].

### 2.1. Ageloxime-D

**Figure 4:**
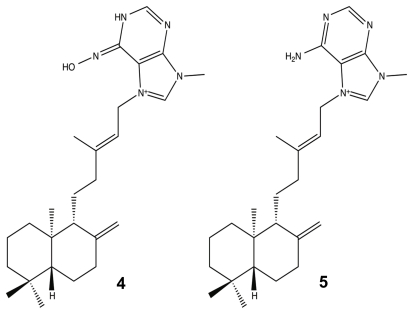
(−)-Ageloxime-D (**4**) and (−)-Agelasine-D (**5**).

The sponge genus *Agelas* (*Agelasidae*) was initially investigated in the mid-1990s because of the potent anti-predatory effects of its bromopyrrole metabolites [71–73,94], but it is now the source of numerous compounds that have immense pharmacologically potential, such as anticancer, antiviral, anti-biofilm, and antibiotic activities [87,95–99]. One class of compounds that has emerged as a unique and potent *Agelas* metabolite is the agelasine class, a diterpenoid [100]. Agelasines can be structurally defined as quaternary adenine salts composed of cyclic diterpene and a 9-methyladeninium moiety [101,102]. Currently, there are at least 12 agelasine derivatives [78,85], and of all these molecules, only one has been shown to have anti-biofilm capabilities without having a bactericidal effect (Figure 4). Last year, T. Hertiani and co-workers reported that the diterpenoid metabolite, (−)-ageloxime-D **4**, was able to inhibit *Staphylococcus epidermidis* biofilm formation without inhibiting bacterial growth, while its parent molecule, (−)-agelasine-D **5**, had the exact opposite effect. In addition to its potential as a biofilm modulator, the oxime, despite only varying from **5** at C-6′, was shown to retain most of its anticancer properties and to have a 10× increase in its antimacrofouling activity [49,78].

### 2.2. Manoalides

**Figure 5:**
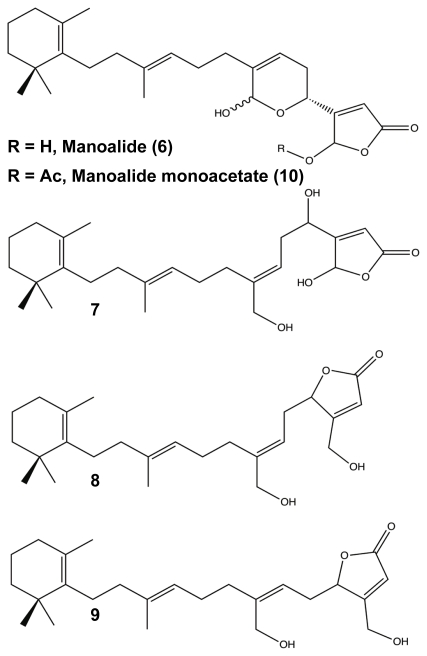
Manolide (**6**), Secomanoalide (**7**), Neomanoalide (*E* (**8**), *Z* (**9**)), and Manoalide Monoacetate (**10**).

Another set of terpenoid compounds that has shown potential as biofilm modulators are also some of the most extensively studied marine sponge metabolites, the manoalides. First identified by Schuler in the early 1980s, manoalide **6** (Figure 5) is a sesterterpenoid from *Luffariella variabilis* with antibiotic and anti-inflammatory abilities [84]. Manoalide acts to block inflammation by irreversibly binding with phospholipase A_2_, an enzyme responsible for converting phospholipids into eicosanoid substrates [103–108]. Shortly after identifying manoalide, Schuler discovered three additional derivatives, secomanoalide **7**, (*E*)-neomanoalide **8**, and (*Z*)-neomanoalide **9** (Figure 5). These derivative compounds and their parent molecule were tested for antibiotic activity and found to be effective against Gram-positive bacteria only [84,109]. In 2007, Skindersoe and co-workers determined that extracts from *L. variabilis* were effective quorum sensing inhibitors (QSI) against both Gram-positive and Gram-negative species [79]. QSIs are molecules that are able to prevent bacterial attachment and biofilm formation by jamming intercellular communication amongst the bacteria. Knowing that manoalides were the major metabolites for *L. variabilis* [110], Skindersoe looked at the QSI activity of manoalide, secomanoalide, and manoalide monoacetate **10** (Figure 5) individually and confirmed that all three compounds were QSIs for both groups of bacteria, attributing the activity to the conserved 2(5*H*)-furanone substituent [79]. While manoalides are Gram-positive bactericides, the combination of the ineffectiveness against Gram-negative bacteria and their role as QSIs provide a strong source for future biofilm modulators, especially against life-threating infections by Gram-negative pathogens (e.g., *Pseudomonas aeruginosa*, *Acinetobacter baumannii*, and *Klebsiella pneumonaie*).

### 2.3. Pyrrole-Imidazole Alkaloids

**Figure 6:**
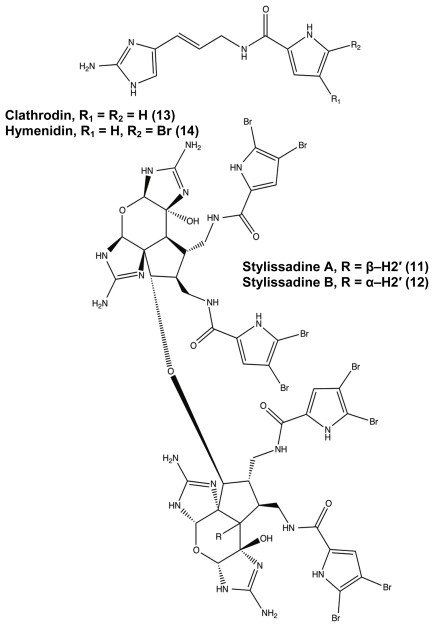
*Agelas* pyrrole-imidazole alkaloids range in complexity from simple oroidin variants (**13**, **14**) to the stylissadines (**11** and **12**).

**Figure 7:**
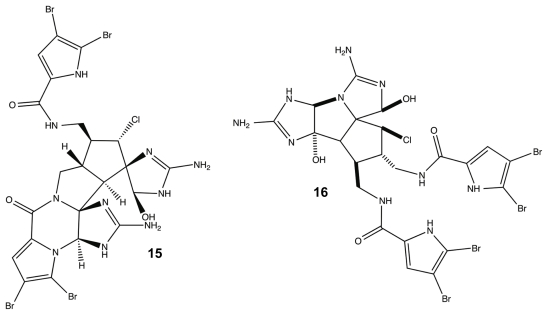
Konbu’acidin (**15**) and Axinellamine (**16**).

While many classes of natural products are shared across multiple species, the pyrrole-imidazole alkaloids have been found exclusively in marine sponges and serve as the major defense metabolites for *Agelasidae*, *Dyctionellidae*, *Hymeniacidonidae*, and *Axinellidae* species [71–73,88,94,111]. In nature, the sessile sponges use these potent molecules as antifeedant defense mechanisms against predatory fish [71,72,88,112,113]. Subsequent investigation into the anti-attachment activity of the PIAs has shown that bromoageliferin and its parent molecule, oroidin, were able to interfere with bacterial attachment of *Rhodospirillum salexigens* and *Vibrio vulnificus* [89,114], but there has been limited research into the anti-biofilm capabilities of other PIAs. This class of molecule can range in complexity from a simple linear oroidin to the 16-stereocenter stylissadines **11** and **12** (Figure 6) [86]. Within the PIAs, oroidin, clathrodin **13**, and hymenidin **14** (Figure 6) are believed to be the basic biosynthetic units for the construction of the larger and more complex congeners, such as konbu’acidin **15** and the axinellamines **16** (Figure 7) [115,116], but one structural feature that is conserved across almost all of the nitrogen-rich PIAs is the presence of the 2-aminoimidazole (2-AI) moiety [111].

**Figure 8:**
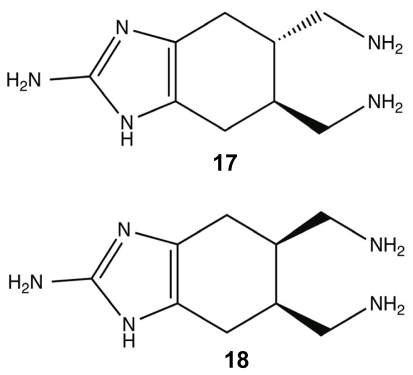
*trans*-bromoageliferin (TAGE) (**17**) and *cis*-bromoageliferin (CAGE) (**18**).

Within most natural product classes, there exists key structural elements or pharmacophores that provide the molecule with its bioactivity and that can be used as the scaffolding for the development of small molecule libraries that can be synthesized in high product yields for further investigation. It was hypothesized that the highly conserved 2-AI groups were principal components of PIA anti-biofilm activity, leading to the development of two bromoageliferin derivatives, TAGE **17** (*trans*-bromoageliferin) and CAGE **18** (*cis*-bromoageliferin) (Figure 8). These 2-AI compounds were assessed for anti-biofilm activity against *Pseudomonas aeruginosa* PAO1 and PA14 biofilms using crystal violet assays and were found to inhibit biofilm formation in a dose-dependent manner (PAO1 IC_50_: 100 μM (TAGE and CAGE); PA14 IC_50_: 190 μM (TAGE) and 180 μM (CAGE)) without demonstrating a bactericidal effect on growth [80]. In addition to inhibiting biofilm formation, TAGE was further able to disperse pre-existing *P. aeruginosa* biofilms with PAO1 EC_50_ of 82 μM and a PA14 EC_50_ of 114 μM [117]. Here, IC_50_ is defined as the concentration required to inhibit 50% biofilm formation; while, EC_50_ is defined as the concentration required to disperse 50% of a preformed biofilm.

**Figure 9:**
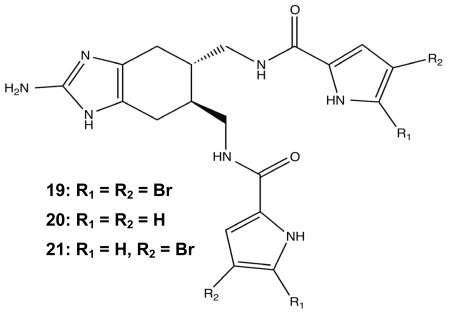
Brominated analogues of TAGE.

To support TAGE’s role as a non-microbicidal biofilm modulator, the phenotypic effects of the molecule were visualized using fluorescence-based experiments with confocal laser scanning microscopy (CLSM) and flow cytometry. One CLSM experiment demonstrated that 100 μM TAGE directly affected the biofilm architecture of static *P. aeruginosa* PAO1 when compared to untreated cultures due to a large decrease in biomass, and the addition of cellular viability staining confirmed that there was not a significant effect on the ratio of living to dead cells in the presence of TAGE. The next fluorescence experiment used flow condition analysis of PAO1:GFP biofilms to validate the crystal violet assays and resulted in a significant decrease in the fluorescence signal in the TAGE samples *versus* the untreated media [117]. Following the success of the 2-AI variants, the next step was to enhance the anti-biofilm properties of TAGE through addition of other important PIA structural motifs. This next generation of bromoageliferin-derived compounds extended the simple structure of TAGE with the addition of brominated acylpyrroles. Three brominated analogues were synthesized, di-brominated **19**, des-brominated **20**, and mono-brominated **21** (Figure 9) and tested for anti-biofilm activity against six γ-proteobacteria species. As the level of bromination increased, the biofilm inhibition activity also increased in comparison to the TAGE scaffold, but the lacked TAGE’s potency against preformed biofilms [117].

**Figure 10:**
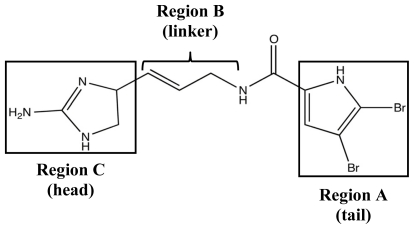
Structure-activity relationship (SAR) design regions for oroidin derivative library.

In the wake of the TAGE triumphs, the 2-AI project was expanded to assess the anti-biofilm efficacy of simpler structures derived from PIAs. The first step was to better analyze the bioactivity of oroidin, the fundamental building block of most PIAs. Following the same assay method used to test the bromoageliferin derivatives, oroidin was found to have very similar biofilm IC_50_ values against PAO1 and PA14 to those of TAGE and CAGE [82,118]. As an added bonus, the results of the oroidin assays supported the previous *R. salexigens* findings and showed that 2-AI activity could have interspecies activity in addition to confirming that the simpler oroidin structure could be used as a modulator scaffold. Therefore, a small chemical library was developed in order to study the structure-activity-relationships (SAR) of oroidin-based analogues as they pertain to anti-biofilm behavior [82,91]. Analogues were designed based on the systematic variation of three regions of the oroidin molecule: the tail, linker, and head groups (Figure 10).

**Figure 11:**
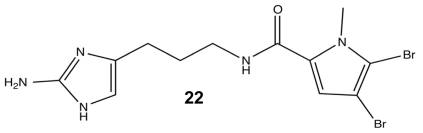
Dihydrosventrin (DHS).

In order to identify the essential structural components for anti-biofilm activity, each modification in the library was evaluated against the PAO1 and PA14 strains. Head group modifications consisted of 2-AI, 2-amino-4-oxoimidazole, imidazole, tryptophan, 2-thioimidazolone, and 2-aminothiazole; while, the tail group was removed or exchanged for a NH pyrrole or *N*-methyl pyrrole with varied degrees of halogenation. The linker region was evaluated based on variation of saturation and aliphatic length, either being truncated or extended by one carbon prior to the amide. While many of these modifications could inhibit biofilm formation, the most potent analogue turned out to be a variant of another known PIA, sventrin. This sventrin congener, known as dihydrosventrin (DHS) **22** (Figure 11), was found to have biofilm inhibition and biofilm dispersion dose-dependent activity against *Acinetobacter baumannii*, different strains of *P. aeruginosa*, and *Bordetella bronchiseptica* RB50 without any microbicidal activity [91]. The SAR studies also provided insight into the importance of the 2-AI head group and which tail group modifications contributed the best anti-biofilm enhancements. The loss of the 2-AI head group on the oroidin derivatives completely destroyed their modulating ability, and as shown by the success of DHS, the best tail groups were those that contained dibromonated *N*-methyl pyrrole moieties.

**Figure 12:**
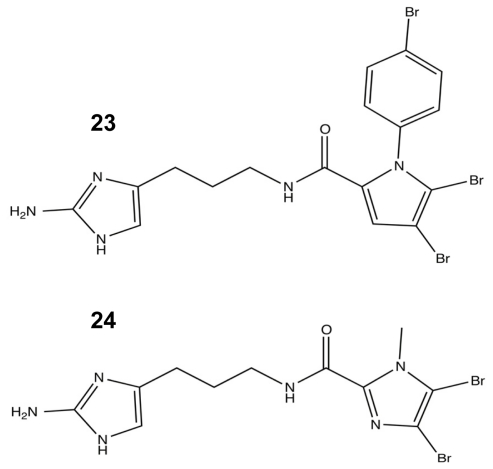
The most (**23**) and least (**24**) successful modifications from the second-generation oroidin SAR.

Armed with the results of the first SAR studies, a second library was developed that targeted the acylation of the pyrrole tail. For this round of analogues, the *N*-heteroaryl position was modified to increase steric bulk, and the importance of the bromopyrrole tail group was further explored using single atom modifications (Figure 12). The *N*-heteroaryl modifications yielded six new and potent biofilm modulators that showed better inhibition and dispersion against *A. baumannii* than their parent molecule, DHS. The best agent was the *p-*bromophenyl addition **23** (Figure 12), which was determined to lack any antibiotic activity. Additional SAR modifications were made to **23** in order to further assess the importance of tail halogenation with the removal of both pyrrole bromines, leaving only the halogenated phenyl group. Without a halogenated pyrrole, the molecule lost a significant amount of its *A. baumannii* anti-biofilm capabilities. In conjunction with halogen SAR studies, the tail ring of DHS was resynthesized with a single atom modification that converted the pyrrole into an imidazole moiety **24** (Figure 12), which caused a drastic drop in activity and reinforced the importance of the dibromonated pyrrole in the tail [91].

**Figure 13:**
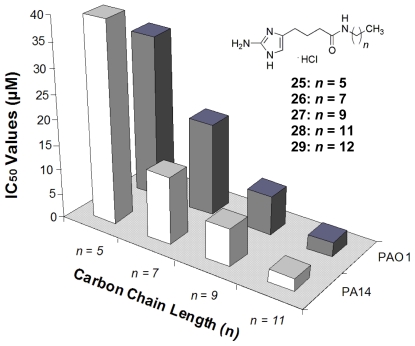
SAR study of the reverse amide tail groups.

While the initial SAR studies of the oroidin analogues provided a detailed understanding of the structural requirements to elicit biofilm modulation activity, the synthetic methods utilized to construct the libraries severely limited the chemical flexibility needed to further compound development. Therefore, two new libraries were devised that focused on the use of alternate connections between the 2-AI head group and the acylpyrrole tail. The first of these chemical libraries stemmed from a reversal of the natural orientation of the amide bond connecting the two portions of the molecule and allowed for more diverse tail group exchange using a variety of commercial amines [119]. Dubbed the “reverse amide” or RA library, only the aliphatic analogues (**25**–**28**) (Figure 13) were found to be viable candidates with biofilm inhibition activity that was four times better than oroidin, and the biofilm inhibition assays showed that the bioactivity of this group was directly related to the length of the aliphatic chain attached to the compound. As the length of the chain increased by five carbons to eleven carbons, the IC_50_ values against *P. aeruginosa* (already in the low μM range) dropped by nearly 20 fold (Figure 13). Additionally, the most active RA molecule, **28**, was found to be a very potent preformed biofilm dispersal agent [119]. To gain better insight into how the length of the aliphatic chain affects anti-biofilm activity, the RA library was expanded to include analogues with up to 18 carbons. When assayed against *P. aeruginosa* PA14, a threshold was determined at a chain length of 13 carbons, **29**, with an IC_50_ value of 729 nM. Beyond that length, the inhibition activity began to drastically decrease [90]. Additional RA modifications that were assessed in the expanded library included altering the location of the amide linkage along the chain, including additional aliphatic chains and the incorporation of a triazole isostere. After assaying these analogues against PA14 and *A. baumannii*, it was evident that the amide bond was not a vital structural element for biofilm activity, but some of these modifications did enhance the molecule’s activity [90].

**Figure 14:**
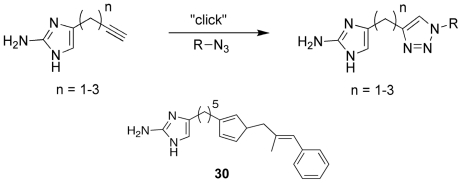
Representation of the “click” reaction used to generate the 2-aminoimidazole/ triazole lead analogue, SPAR.

The second 2-AI library that was developed looked to increase the range of available analogues by utilizing the compatibility of the click reaction with a diverse array of substrates to replace the pyrrole tail group. A library of 2-aminoimidazole/triazole (2-AIT) compounds was generated through a click reaction involving an unprotected 2-AI alkyne species with varying linker lengths and an azide bearing the tail group of interest (Figure 14) [93]. From the initial biofilm activity screens against *P. aeruginosa*, *A. baumannii*, and *B. bronchiseptica* RB50, a single active agent was discovered that possessed a substituted unsaturated aryl group. Retaining the aryl pendant group, additional analogues were synthesized that examined the effects of extending the 2-AI linker region. This round of assays yielded one of the most potent anti-biofilm compounds to date, **30** (Figure 14). Possessing strong inhibitory and dispersal capabilities across all assayed Gram-positive and Gram-negative bacterial species without any antimicrobial activity, **30** established a highly successful template for further 2-AIT derivations [93]. The current research into the anti-biofilm properties of the 2-AI analogues has shown that, not only is the oroidin class a member of a select group of natural products with the ability to modulate biofilm formation, but it also directly affects the biofilm phenotype across multiple species without antibacterial or antimicrobial activity that could lead to the development of resistance phenotypes.

## 3. Anti-Biofilm Agents Clinical and Industrial Status

Therapeutic interest in sponge metabolites has been active since Bergmann’s research during the early twentieth century lead to the development of antileukemia drug ara-C [120,121]. Over the decades, marine sponges have yielded a diverse array of molecules that have been linked to anti-inflammatory, antitumor, antiviral, antiprotozoal, and antibiotic activities [13,122]. Now, sponges have become a source of non-microbicidal biofilm modulators that could help rescue the medical field from the increasing threat of MDR infections. To date, very little research has furthered the anti-biofilm applications of the terpene compounds, ageloxime-D or the manoalides, but extensive pre-clinical studies have been performed on the 2-aminoimidazole libraries.

**Figure 15:**
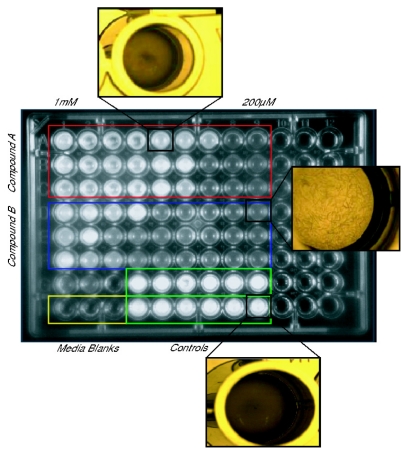
Example of *C. elegans* toxicity assay used to assess the RA and 2-aminoimidazole/triazole (2-AIT) lead compounds [92].

The native toxicity of the PIAs serves as a potent defense mechanism for sponges, and it also acts as a major hurdle that must be overcome before these compounds can be used from medical treatment. The MTT evaluation of the first generation of bromoageliferin derivatives, TAGE and CAGE, against GH4C1 rat pituitary and N2A mouse neuroblastoma cell lines showed that the 2-AI analogues lacked the mammalian cytotoxicity of their parent molecule [117,123]. Subsequent toxicological screens of the RA and 2-AIT libraries using *Caenorhabditis elegans* further supported the negligible toxicity of these compounds with minimal exposure thresholds of nearly 800 μM or higher in many cases five to ten times greater than the compound’s inhibition concentrations (Figure 15) [92].

**Figure 16:**
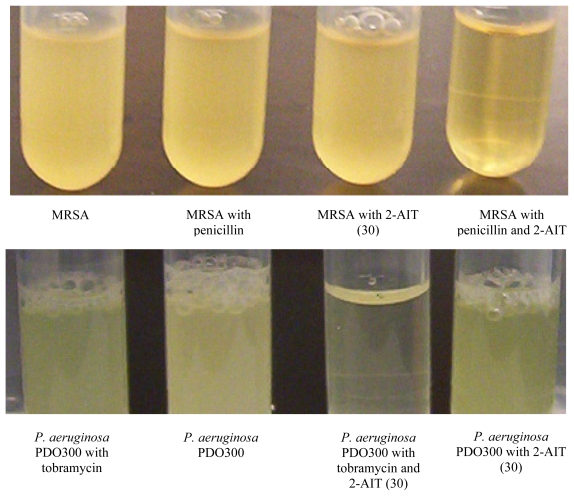
Adjuvant activity assay with the lead 2-AIT, SPAR (30), and tobramycin against *Pseudomonas aeruginosa* PDO300. Shows that tobramycin and 2-AIT alone are not effective against PDO300, but combined the 2-AIT makes PDO300 more susceptible to tobramycin [83].

While the broad spectrum and potent anti-biofilm properties of the 2-AI molecules are enough to merit further study, recent work with the lead 2-AIT, SPAR, has shown the promise of these molecules as adjuvants for conventional antibiotic therapy [83]. When combined with conventional antibiotics, the 2-AIT conjugate has proven to be able to resensitize previously resistant bacterial strains to the antibiotic, allowing the drug to resume its activity. In particular, this compound was able to resensitize drug-resistant methicillin-resistant *Staphylococcus aureus* (MRSA) and multi-drug resistant *Acinetobacter baumannii* (MDRAB), both extremely dangerous causative agents of nosocomial infections, to many of the antibiotics used to treat their non-resistant counterparts, such as penicillin G, tetracycline, and imipenem (Figure 16) [83].

**Figure 17:**
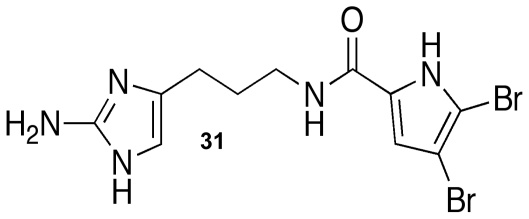
Dihydrooroidin (DHO).

In addition to a plethora of pharmacological applications, marine sponge metabolites have been actively pursued for their antifouling applications [43,44,49]. Many of the terpene and pyrrole-imidazole alkaloids have proven to have antifouling capabilities against both microfouling and macrofouling organisms, but many of these compounds are either toxic or have limited industrial applications due to formulation difficulties. Of the biofilm modulators reported in this review, the only compound that has been tested directly for marine antifouling activity has been the oroidin analogue, dihydrooroidin (DHO) **31** (Figure 17). DHO is an easily synthesized oroidin variant [118] that was an active inhibitor of biofilm formation against the bacterial fouling agent, *Halomonas pacifica*. Based on its success against *H. pacifica*, DHO was combined with marine-based paint and subjected to mesocosm tank trials. After three weeks in the tanks, the DHO paint had 125% less biomass than the paint-only controls. These trials showed that DHO was a viable antifouling agent and was still active after three weeks in the tank. Since PIAs are notorious toxins, DHO toxicity was evaluated using the same mammalian cytotoxicity assays used to evaluate TAGE and was found to be nontoxic up to six times the dosage used in the paint trials [81,117]. The combination of their medicinal and antifouling applications have proven the power and far-reaching potential of the 2-aminoimidazole compounds to help overcome many of the complications caused by biofilm-forming bacteria.

## 4. Future Perspectives

As we advance our knowledge of how to design potent anti-biofilm molecules, a few issues have become clear. First, we need to understand the basic mechanistic aspects of how these molecules exert their activity. These molecules have the ability to provide tools to deconvolute bacterial signaling pathways that are both conserved and unique to Gram-positive and Gram-negative bacteria. Second, these compounds need to be evaluated in various animal models of infection. As a therapeutic strategy, these compounds will mostly likely serve as adjuvants to conventional antibiotics, and there are dosing and pharmacokinetic/pharmacodynamic (PK/PD) issues that must be optimized between the antibiotic and anti-biofilm agent. Third, new molecular classes should be investigated to expand our repertoire of anti-biofilm agents. To this end, we recently started to investigate the flustramine family of natural products to probe indole signaling, a pathway proposed along with quorum sensing AI-2 to be a universal interspecies signal [124–126]. Fourth, now that we have potent anti-biofilm molecules, the various *in vitro* biofilm assays need to be compared to *in vivo* outcomes.

Finally, further toxicity tests need to be established, especially with regards to the effects of anti-biofilm compounds on commensal bacteria in their native host (such as gut bacteria). Before advancing to costly animal models, it has been shown that simple nematode models are a good source of preliminary toxicological analysis, providing a high-throughput platform for evaluating cytotoxicity and host neurological, developmental, and immune dose-responses [127–133]. Major advantages of using *C. elegans* for toxicity studies include its completed genome, short lifespan, and simple physiology and have lead to the development of rapid assays that can measure dosage effects on gene expression and aid in determining sublethal dosages using a variety of indicators, such as RNA/DNA [128,134–137]. As a preliminary “curing” study, the use of *C. elegans* offers a simple, high-throughput method for evaluating the activity of these compounds against an active infection, providing insight into both host and pathogen *in vivo* dose responses [135,137–139].
